# Miura-origami-inspired electret/triboelectric power generator for wearable energy harvesting with water-proof capability

**DOI:** 10.1038/s41378-020-0163-1

**Published:** 2020-08-10

**Authors:** Kai Tao, Haiping Yi, Yang Yang, Lihua Tang, Zhaoshu Yang, Jin Wu, Honglong Chang, Weizheng Yuan

**Affiliations:** 1 Research & Development Institute in Shenzhen, School of Mechanical Engineering, Northwestern Polytechnical University, 518057 Shenzhen, People’s Republic of China; 2 Department of Mechanical Engineering, University of Auckland, 20 Symonds Street, Auckland, 1010 New Zealand; 3 State Key Laboratory of Optoelectronic Materials and Technologies, the Guangdong Province Key Laboratory of Display Material and Technology, School of Electronics and Information Technology, Sun Yat-sen University, 510275 Guangzhou, People’s Republic of China

**Keywords:** Electrical and electronic engineering, Electronic devices

## Abstract

One of the critical issues for electret/triboelectric devices is the poor charge viability and stability in humid environments. Herein, we propose a new origami-inspired “W-tube”-shaped triboelectric nanogenerator (W-TENG) with two thin-film electrets folded based on Miura-origami. The Miura-origami fold is capable of transforming flat materials with large surface areas into reduced and compressed complex 3D structures with parallelogram tessellations. The triboelectric power generation components can thus be hermetically sealed inside the “W-tube” to avoid contact with the external humid environment. Furthermore, the elastic nature of the Miura-origami fold endows the proposed W-TENG device with excellent deformability, flexibility, and stretchability. Therefore, it is capable of harvesting kinetic energy from various directions and forms of movement, including horizontal pressing, vertical tapping, and lateral bending. The compact, light weight, and self-rebounding properties of the origami structure also make it convenient for integration into wearable devices. Various parameters of the W-TENG are intensively investigated, including the number of power generation units, original height of the device, acceleration magnitude, excitation direction, and water-proof capability. Triggered by hand tapping impulse excitation in the horizontal and vertical directions, the instantaneous open-circuit voltages can reach 791 V and 116 V with remarkable optimum powers of 691 μW at 50 MΩ and 220 μW at 35 MΩ, respectively. The outcomes of this work demonstrate the fusion of the ancient art of origami, material science, and energy conversion techniques to realize flexible, multifunctional, and water-proof TENG devices.

## Wearable electronics: Folding the way to flexible a power supply

Researchers have used techniques from origami to engineer a flexible, resilient, water-proof power generator, which can be used with wearable devices. The device, designed by a team led by Kai Tao of China’s Northwestern Polytechnical University, consists of two thin films of dielectric material, which can generate power when deformed. This structure is then folded in a Miura fold, a pattern which compresses a large surface area into a smaller area. The resulting spring-like makes the device return to its original shape after deformation while also offering compactness and flexibility together. The device generated power when tapped or bent, and the origami structure hermetically sealed the components, making the generator water-proof. With this combination of origami and material science, researchers can design and build flexible energy-harvesting devices to power wearable devices or other flexible electronics.

## Introduction

Recent advances in the internet of things (IoT) and flexible electronics have given rise to the rapid expansion of flexible sensing devices, roll-up displays, soft robots, and bioinspired artificial skins^1–4^. Meanwhile, these low-power electronic devices pose a challenge to cheap, flexible, portable, and sustainable energy resources^5–8^. Fundamentally, these power sources should be compact, light weight, shape adaptive with good wearing comfort, comparably stretchable, and flexible. In addition, the devices should have robust mechanical durability to sustain cyclic pressing, bending, stretching, and folding processes without deterioration of their performance in practical applications.

Energy harvesting refers to capturing and transforming environmental wasted energy into electrical energy as a secondary power source for IoT devices and flexible electronics^9–12^. There are mainly five energy source candidates for energy harvesting in the environment, light energy, mechanical vibrations, thermal gradients, radio frequency (RF) waves, and acoustic energy. Mechanical vibrations derived from machine and structure vibrations, human motion, and air/water flows ubiquitously exist in ambient environments^13–15^. Generally, mechanical vibration energy can be converted to electrical energy through piezoelectric^16–20^, electromagnetic^21–24^, electrostatic^25–30^, magnetostrictive^31,32^, and triboelectric mechanisms^33–37^. Triboelectric nanogenerators (TENGs) based on the coupling of contact electrification and electrostatic induction have been proven to be an efficient technology for converting mechanical energy to electricity. TENGs have unique features in terms of high-energy conversion efficiency, diverse material selection, cost effectiveness, simple design, and ease of fabrication^38–40^. Therefore, TENGs have tremendous potential to meet the energy supply demand arising from the rapid growth of wearable and flexible electronics.

Origami, an ancient art of paper folding with a history spanning over 1000 years, is capable of devising ingenious patterns relying only on paper itself. Structures inspired by origami possess many advantages in terms of light weight, great flexibility, excellent shape adaptability, and extreme simplicity. Therefore, attempts to adopt origami art in the design of TENGs appear to be promising for wearable and flexible electronic applications. Peng Bai et al.^41^ proposed a Kapton-film-based origami TENG with a zigzag structure. Five energy generation units were bonded onto a single flexible substrate with an ultralight weight of 7 g. Hengyu Guo et al.^42^ proposed an ultralight-weight cut-paper-based rhombic-shaped TENG. The TENG power generation unit and a supercapacitor energy storage unit can be integrated together. Yang et al.^43^ developed a type of slinky and doodlebug-shaped TENG that operates in a single-electrode contact mode. Kai Tao et al.^44^ further developed an electret-based origami TENG on a liquid crystal polymer substrate with a double-helix spring structure. The versatile origami design makes the device applicable in both wearable device and wave energy-harvesting scenarios. Feng et al.^45^ proposed an arc-shaped paper-based TENG with gum wrappers and conductive Al layers for self-powered anticorrosion and antifouling applications. However, none of the previous studies attempted to exploit origami TENGs with water-proof capabilities in multiple operation modes.

This paper proposes a new type of “W-tube”-shaped triboelectric nanogenerator (W-TENG) with two pieces of thin-film electrets folded based on Miura-origami. By employing Miura-origami, the proposed W-TENG exhibits several advantages:

- A self-sustained spring structure is readily obtained by Miura-origami. The W-TENG is able to bounce back to its original state without auxiliary supporting structures, making the whole structure compact, light weight, flexible, and deformable^46,47^. The facile fabrication process involves easy low-cost commercial production.
- To improve the charge viability and stability in humid environments, the power generation components of the W-TENG device, including the thin-film electrets and copper contact electrodes, are hermetically sealed inside the “W-tube”, avoiding corrosion and deterioration arising from external environments.
- The three-dimensional (3D) “W-tube” TENG is formed by face-to-face assembly of two pieces of 2D Miura-origami-shaped strips. The zigzag deployment makes the proposed W-TENG capable of harvesting kinetic energy from various directions and forms of motion, including horizontal pressing, vertical tapping, and lateral bending.
- A high-performance TENG can be readily obtained by using the stacked multilayer Miura-origami fold structure. The corona discharge process is further employed to preimplant charge into the electrets to maximize the charge trap density. Both the electrostatic induction and the contact electrification are significantly amplified by the increased electrode areas and preimplant charges.

With Miura-origami folding, a W-TENG with a size of 14.5 × 5.8 × 3 cm^3^ is fabricated and constructed with eight power generation units connected in parallel. Triggered by gentle hand tapping in the horizontal and vertical directions, instantaneous open-circuit voltages of 791 V and 116 V with remarkable output powers of 691 μW and 220 μW are obtained, respectively. The flexible and light-weight W-TENG is suitable for flexible electronics and biomechanical energy-harvesting applications. Furthermore, the water-proof property of the W-TENG device further broadens its application scenarios in humid environments.

## Experimental methods

Miura-origami folding, invented by Japanese astrophysicist Koryo Miura, is a rigid form of the flat-origami-folding technique that allows one to transform flat materials with a large surface area into reduced and compressed complex 3D structures through a tessellated crease pattern made of repeating parallelograms. Each parallelogram unit remains flat and rigid throughout the origami process when the continuous folding is carried out one-fold after another. Remarkable mechanical and material properties can be obtained, with greater compressibility, rigidity, stiffness, and contractile ability, known as a negative Poisson’s ratio. Herein, a type of W-TENG based on Miura-origami folding is fabricated and characterized for multifunctional energy harvesting with water-proof capability, as shown in the following sections.

**Fig. 1:**
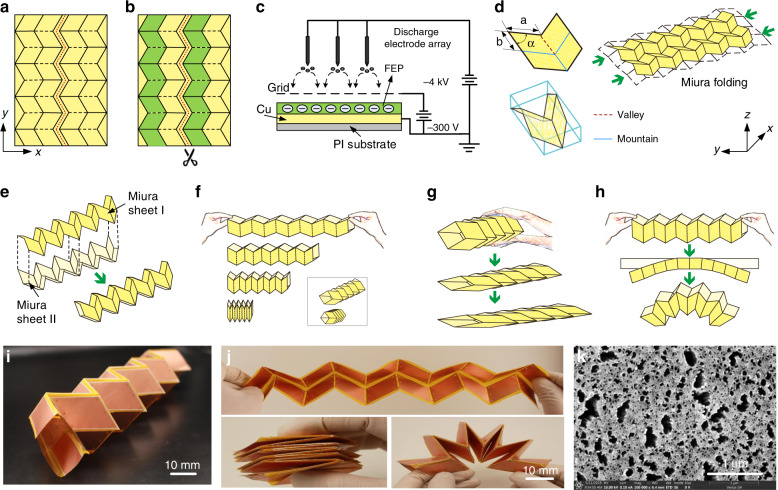
Configuration and formation of proposed Miura-origami-inspired W-TENG device. **a** A crease pattern of a Miura-origami tessellation with an array of parallelograms; **b** thin-film FEP electrets are attached to every other zigzag path of the Miura-patterned FPCB; **c** a corona discharge setup is used to preimplant charges into the thin-film FEP electrets; **d** simultaneous and homogenous zigzag folding process in orthogonal directions; **e** assembly process of the proposed “W-tube”-shaped TENG structure with two pieces of Miura-origami sheets; **f**–**h** three operation modes of the proposed W-TENG device in three axial directions, including horizontal pressing, vertical tapping, and lateral bending motions; **i** photograph of the fabricated W-TENG prototype with eight power generation units connected in parallel; **j** optical photographs of the fabricated W-TENG prototype in stretched, compressed, and bent states; **k** SEM images of FEP electrets with nanostructured pillars fabricated by an ion beam etching (IBM) process

Figure 1a shows the crease patterns of the Miura-origami fold, with an array of parallelograms used to form a tessellation of the surface. Each parallelogram translates to its neighboring unit by mirror reflection along the horizontal crease lines, forming zigzag crease paths in the vertical direction. The solid and dashed lines denote mountain and valley folds, respectively. The mountain folds and valley folds alternate from one zigzag path to the next. Therefore, the planar sheet can be packed into a compact shape by pressing the two ends together and likewise unpacked by pulling on its opposite ends.

Figure 1b shows that the thin-film fluorinated ethylene propylene (FEP) electrets are attached to every other zigzag path. The covered and uncovered areas denoted by yellow and green colors represent the contact triboelectric materials and electrodes of the W-TENG device, respectively. Figure 1c shows the corona discharge process with a triode-needle-grid setup. A high-potential gradient can ionize the air around the tip of the needle. The ionized particles are then driven into the below thin-film FEP electrets by the electric field between the grid and substrate. The charges implanted in the thin-film electrets maintain a quasi-permanent bias voltage, which can form a permanent electric field around the device for years.

Figure 1d shows the simultaneous and homogenous zigzag folding process in the orthogonal direction. The folding property is determined by the design parameters of each parallelogram, i.e., the two side lengths and the intersection angle. Figure 1e shows the assembly process of the W-TENG structure by mirror-image sticking of two pieces of Miura-origami sheets together. The edges of the sheet are coated with standard paper adhesive. The hermitically sealed “W-tube” structure is therefore constructed by the aligned assemblage with the adjacent facets of two Miura-origami sheets adhered together.

Figures 1f–h demonstrate the three operation modes of the proposed W-TENG device in three axial directions, including horizontal pressing, vertical tapping, and lateral bending motions. The zigzag deployment of the Miura-origami fold ensures that the contact material of each parallelogram is different from that of the parallelogram next to it. Therefore, triboelectrification can occur when any two electrodes become in contact with each other. In addition, the inherent nature of the Miura-origami fold endows the proposed W-TENG device with excellent flexibility and stretchability. It is capable of bouncing back to its original state based on the restoring force of the origami spring itself, without relying on other supporting structures. These unique features make it very applicable to flexible electronics and biomechanical energy-harvesting applications.

Figure 1i shows the fabricated W-TENG prototype. The Miura-origami sheets are created from double-side copper-coated 14.18 g/cm^2^ flexible printed circuit board (FPCB) with an array of parallelogram units with a length of 30 mm, a width of 25 mm and an intersection angle of 60°. A “W-tube”-shaped TENG structure with a size of 14.5 × 5.8 × 3 cm^3^ was fabricated and constructed with eight power generation units connected in parallel. Figure 1j shows the stretched, compressed and bent states of the fabricated W-TENG prototype. The proposed W-TENG prototype can be packed into a stacked flat and compact disk with thickness reflecting only the thickness of the folded W-TENG material. The large tensile and compressive deformation properties demonstrate that the W-TENG prototype has good ductility, stretchability and deformability. Figure 1k shows SEM images of thin-film FEP electrets with nanostructured pillars fabricated by an ion beam etching (IBM) process, which are beneficial for contact electrification.

**Fig. 2:**
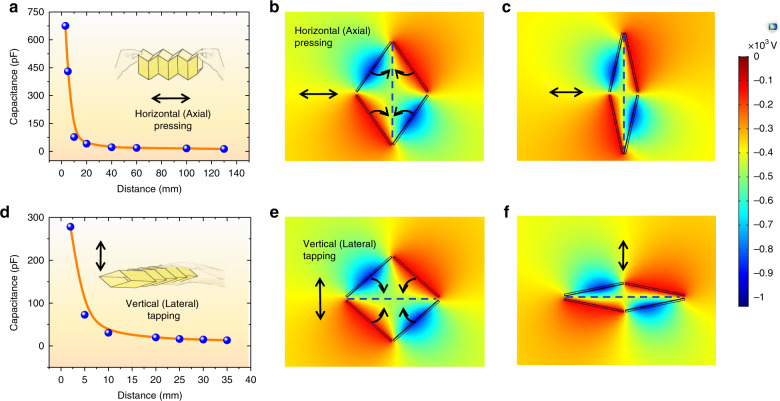
Working principle of the power generation unit in the horizontal axial and vertical lateral directions. **a** Capacitance variation versus length of the W-TENG; **b**, **c** contour snapshots of the electric field distribution in stretched and compressed states in the horizontal axial direction; **d** capacitance variation versus height of the W-TENG; **e**, **f** contour snapshots of the electric field distribution in stretched and compressed states in the vertical lateral direction

Figure 2 shows the working principle of a single power generation unit in the horizontal axial and vertical lateral directions. Figure 2a shows the capacitance variation versus the length of the W-TENG in the horizontal axial direction. Both ends of the W-TENG are stuck together by hand. The capacitance variation is monitored in real time by an impedance analyzer (Applent AT811). By connecting eight power generation units in parallel, it is seen that the capacitance changes from 15 to 675 pF when the length of the W-TENG varies from 125 to 3 mm. In other words, the capacitance can be increased by 44 times as the length of the W-TENG is decreased by 97.6%, demonstrating a very large capacitance change and excellent compression capability of the W-TENG device. The capacitance dramatically increases when the length is below 20 mm. This is because the capacitance change is ten times higher when the length of the W-TENG changes from 20 to 3 mm than that when it varies from 120 to 20 mm. The output power of an electret/triboelectric device is quadratically proportional to the ratio of the capacitance change. Therefore, a large output power can be readily achieved with such great capacitance variations.

Contours of the potential gradient and electric field distribution for different deformations of the W-TENG device can be estimated by COMSOL Multiphysics simulation. Supplementary material Video S1 shows the dynamic contours for different deformations obtained by COMSOL. Figure 2b, c show contour snapshots of the electric field distribution in the stretched and compressed states, respectively. The intensity of the electric field dramatically increases when the two electrodes become closer. Correspondingly, Fig. 2d–f demonstrate the capacitance and electric field contour variations versus the displacement of the W-TENG in the vertical lateral direction. The height of the W-TENG decreases from 35 to 3 mm, corresponding to a displacement change percentage of 91.4%. The capacitance increases from 13 to 280 pF, corresponding to a capacitance change ratio of 20.5 times. In the vertical lateral mode, the electret material contacts the other neighboring electrode, different from that in the horizontal axial mode.

## Results and discussions

The fabricated W-TENG prototype is characterized under sinusoidal and impulse excitations in both the horizontal and vertical directions. The measurement setup consists of mainly an electrodynamic shaker, a function generator, a voltage amplifier, an accelerometer and a data acquisition system. The W-TENG prototype is fixed between two parallel plates with the top plate fixed and the bottom plate excited by the shaker. The movement of the bottom plate is monitored by the accelerometer in real time. The output data are collected by a data acquisition system (DAQ NI USB-6289 M series), which is connected and controlled by a laptop. In the current study, various parameters have been intensively investigated and optimized, including the number of power generation units, original height of the W-TENG device, external excitation acceleration, excitation direction, wearable conditions, and water-proof capability under moisture environments.

### Output performance with different original heights

**Fig. 3:**
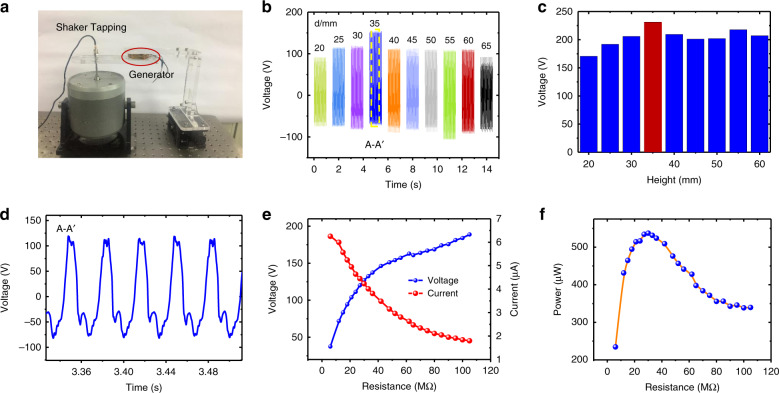
Output performance of the W-TENG with original height ranging from 20 to 65 mm under the horizontal axial pressing mode with eight power generation units electrically connected in parallel. **a** Sinusoidal excitation experimental setup with the W-TENG device sandwiched between two parallel plates; **b**, **c** output voltages for different original heights of the W-TENG; **d** close-up view of the output voltage waveform when the original height is set to 35mm; **e** output voltage and current with varying load resistance at the original height of 35mm; **f** output power with varying load resistance at the original height of 35mm

Figure 3 shows the output performance of the W-TENG with different original heights under the horizontal axial pressing mode with eight power generation units electronically connected in parallel. The fabricated W-TENG is sandwiched between two parallel plates. One end is fixed to the shaker, while the other end is bonded to a positioning stage whose height can be precisely controlled. The sinusoidal excitation experimental setup is shown in Fig. 3a. The excitation acceleration is set to 19.6 m/s^2^. Figure 3b, c show the time-domain output voltages for different original heights ranging from 20 to 65 mm under a load condition of 25 MΩ. The output performance is determined by the spacing variation and contact condition of the different power generation units. The performance is highly dependent on the device original height and external excitation displacement. The maximum output performance is usually obtained when the capacitance variation reaches the maximum value at the closest positions of the electrodes. The material height of the eight power generation units is ~10 mm. The maximum displacement of our shaker is up to 10 mm. Therefore, 20 mm is the minimum height that can be achieved. Otherwise, the prototype cannot be compressed or is vulnerable to damage due to the increased stiffness with the small thickness of the W-TENG device. The optimum performance is achieved with an original height of ~35 mm. Figure 3d shows a close-up view of the output voltage waveform, which is not the standard sinusoid. This is due to the uneven distance variations and contact conditions among different power generation layers. Figure 3e shows the output voltages and currents for different load resistances in the range of 3–105 MΩ. An output voltage of 188 V is obtained at a load resistance of 105 MΩ, which is approximately equal to the open-circuit voltage condition. An output current of 6.25 μA is obtained at a load resistance of 6 MΩ. Figure 3f shows that the maximum output power of 537 μW is obtained at the optimum load resistance of 30 MΩ.

### Output performance with different numbers of power generation units

**Fig. 4:**
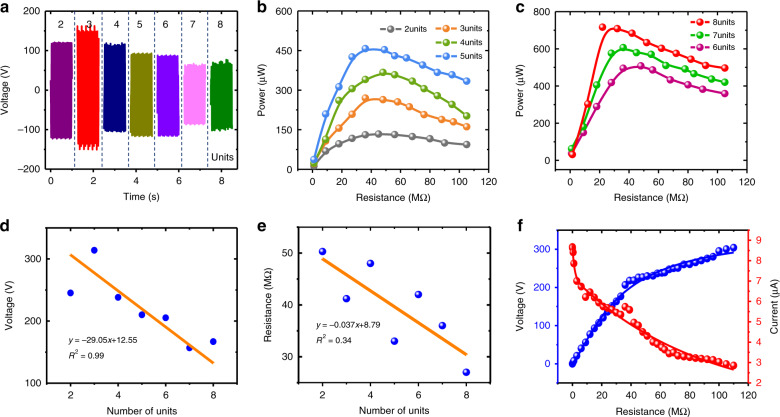
Output performance of the W-TENG versus number of power generation units in the range of 2–8 at an acceleration of 19.6 m/s^2^. **a** Time-domain output voltages with different numbers of power generation units of the W-TENG under 50 MΩ; **b**, **c** output powers with different numbers of power generation units versus load resistance optimization in the range of 3–105 MΩ; **d** open-circuit voltage versus number of power generation units; **e** optimum load resistance versus number of power generation units; **f** output voltage and current variations with load resistance ranging from 100 kΩ to 110 MΩ for eight power generation units

Figure 4a shows the time-domain output voltages of the W-TENG with a power generation unit number in the range of 2–8 and an original height of 25 mm at an acceleration of 19.6 m/s^2^ under the horizontal axial pressing mode. Figure 4b, c show output powers at different load resistances of 3–105 MΩ with power generation unit number in the range of 2–5 and 6–8 units, respectively. It can be clearly seen that the overall output power of the W-TENG has a significantly positive correlation with the number of power generation units employed. With an increase in the number of power generation units from 2 to 8, the output power is tremendously enhanced from 147 to 716 μW. In contrast, Fig. 4d shows that the open-circuit voltage of the TENG demonstrates a clearly negative correlation with the number of power generation units. This is mainly because the power generation contribution with increasing unit number is largely reflected in the increase in the output current. This can be further verified by Fig. 4e, which shows that the optimum load resistance has a downward trend with increasing number of power generation units. Fundamentally, the maximum output power can only be reached when the external load equals the internal impedance of the generator^48^. In general, the internal capacitance increases with increasing number of power generation units. Therefore, a lower optimum load resistance is obtained with increasing number of power generation units according to the impedance expression *R* ∝ 1/*jωC* ^49^. Figure 4f shows the output voltages and currents for different load resistances ranging from 100 kΩ to 110 MΩ. The maximum voltage of 305 V and current of 8.6 μA are obtained with load resistances of 110 MΩ and 100 kΩ, respectively.

### Output performance with different excitation accelerations

**Fig. 5:**
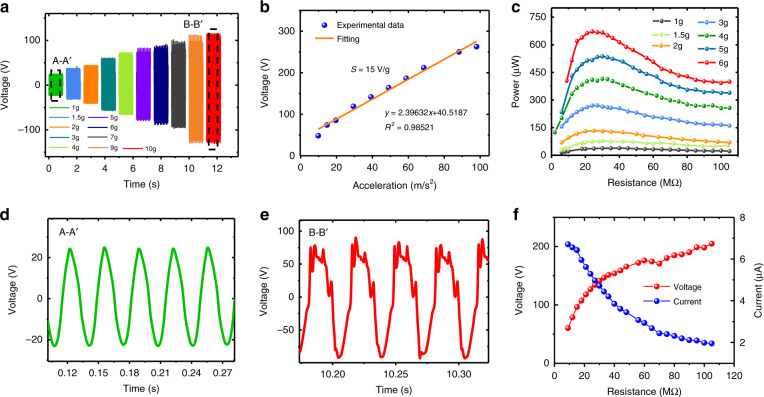
Output performance characterization of the W-TENG versus different excitation accelerations. **a** Voltage waveforms with different excitation accelerations ranging from 9.8 to 98 m/s^2^ under a load resistance of 30 MΩ; **b** voltage amplitude variation with acceleration under a load resistance of 30 MΩ; **c** output power for different excitation accelerations ranging from 9.8 to 98m/s^2^ versus load resistance ranging from 3 to 105 MΩ; **d** enlarged view of open-circuit voltage waveforms at an acceleration of 9.8m/s^2^; **e** enlarged view of open-circuit voltage waveforms at an acceleration of 98m/s^2^; **f** output voltage and current variations with load resistance ranging from 3 to 105 MΩ at an acceleration of 58.8m/s^2^

Figure 5 shows the output performances of the W-TENG for different excitation accelerations with an original height of 35 mm and eight units electrically connected in parallel under the horizontal axial pressing mode. Figure 5a, b show the output voltage waveform and amplitude variations of the W-TENG with excitation acceleration ranging from 9.8 to 98 m/s^2^ at a load resistance of 30 MΩ. The output voltage increases from 48 to 262 V when the acceleration changes from 9.8 to 98 m/s^2^. It can be seen that the output voltage is quasi-linearly positively correlated with the excitation acceleration, with a slope of 1.5 V/(m/s^2^). This indicates that the W-TENG has the potential to be developed as a self-powered force/acceleration sensor. Figure 5c shows the output powers for different excitation accelerations ranging from 9.8 to 58.8 m/s^2^ with varying load resistances ranging from 3 to 110 MΩ. The maximum output power of 670 μW is achieved at the optimum load resistance of 25 MΩ and an acceleration of 58.8 m/s^2^. The optimum load resistance decreases with increasing acceleration. This shows a similar phenomenon to the trend with the number of power generation units in the previous discussion. Figure 5d, e show close-up views of the open-circuit voltage waveforms under accelerations of 9.8 m/s^2^ and 98 m/s^2^, respectively. The waveform of the open-circuit voltage suffers from serious deformations at the higher excitation acceleration of 98 m/s^2^. This is due to the unstable capacitance variation of the origami structure when it is squeezed into a very low height. It cannot bounce back to its original height as fast as in low-acceleration conditions. Figure 5f shows the output voltage and current variations with load resistance ranging from 3 to 110 MΩ at an acceleration of 58.8 m/s^2^. The maximum output voltage of 205 V and current of 6.7 μA are obtained with load resistances of 110 MΩ and 100 kΩ, respectively.

### Output performance for different direction excitations

**Fig. 6:**
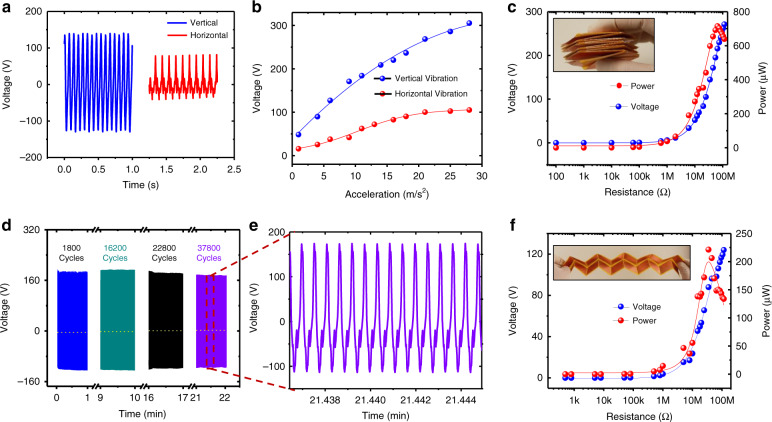
Output performance characterizations of the W-TENG for sinusoidal excitations in the horizontal axial and vertical lateral directions. **a** Open-circuit voltage waveforms for the two different directions at 6m/s^2^; **b** output voltage amplitude variations for the two directions with excitation acceleration; **c** output voltage and power optimizations for the horizontal axial direction with different load resistances ranging from 3 to 105 MΩ; **d** output performance stability of the W-TENG during continuous operation over ~37,800 cycles; **e** enlarged view of output voltage waveforms; **f** output voltage and power optimizations with different load resistances ranging from 3 to 105 MΩ for the vertical lateral direction

Figure 6 shows the output performance characterizations of the W-TENG for excitations in the horizontal axial and vertical lateral directions with original heights of 35 mm and 30 mm, respectively. Figure 6a shows the open-circuit voltage waveforms for the two different directions at an acceleration of 6 m/s^2^. The output voltage for the horizontal axial direction is much larger than that for the vertical lateral direction. Figure 6b shows the output voltage amplitude variations for the two directions with excitation acceleration ranging from 2 to 28 m/s^2^. With increasing acceleration, the growth rate of the output voltage decreases, exhibiting a nonlinear and spring hardening relationship. This is mainly due to the increase in the spring stiffness under high-deformation conditions of the Miura-origami structure. Figure 6c, f show the output voltage and power optimizations for impulse excitation by hand tapping in the horizontal axial and vertical lateral directions at different load resistances ranging from 3 to 105 MΩ. The instantaneous open-circuit voltages for the horizontal and vertical directions can reach 791 V and 116 V with optimum powers of 691 μW at 50 MΩ and 220 μW at 35 MΩ, respectively. Figure 6d, e show the output performance stability and an enlarged view of the voltage waveforms of the W-TENG during continuous operation for ~37,800 cycles. Slight deterioration of the performance is observed after long-term operation, indicating the stable performance of the fabricated W-TENG device.

### Output characterization for impulse excitation by human motions

**Fig. 7:**
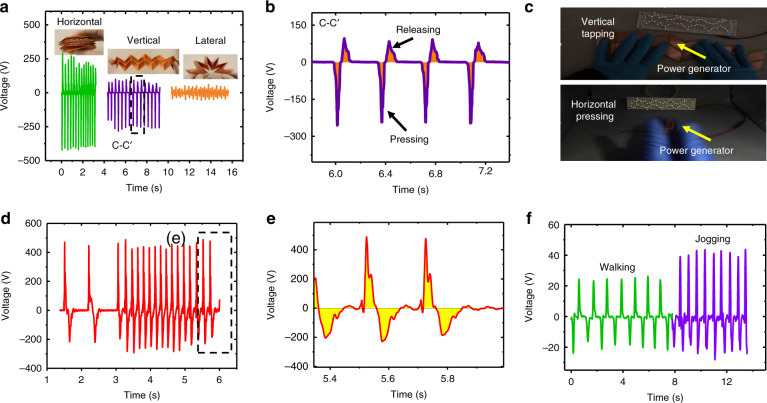
Impulse excitation characterization of the W-TENG with eight power generation units connected in parallel for human motion. **a** Comparisons of the output voltage waveforms for different deformation forms, such as horizontal pressing, vertical tapping and lateral bending; **b** Enlarged view of the open-circuit voltage waveforms for the vertical tapping motion; **c** snapshots of a power generation demonstration in which LEDs are lit up by horizontal pressing and vertical tapping; **d** output voltage waveforms for different hand pressing frequencies in the horizontal axial direction; **e** enlarged view of output voltage waveforms; **f** output performance demonstration in which the W-TENG is installed in shoes during walking and jogging

Figure 7 shows the output performance characterization of the W-TENG under impulse excitation by human motions. Figure 7a shows comparisons of the output voltage waveforms for different deformation forms, such as horizontal pressing, vertical tapping, and lateral bending. The Miura-origami natural property makes the versatile W-TENG extremely flexible under multidirectional excitations. Figure 7b shows enlarged open-circuit voltage waveforms for the vertical tapping motion. The filled orange parts denote the energy collected during the pressing and releasing process. It can be clearly observed that the peak of the output performance is much larger than that obtained by shaker tapping. This is because the impact force and speed of impulse excitation by hand tapping is much larger than those by shaker tapping, leading to a larger displacement and faster movement of the origami device. Furthermore, the hand pressing and recovery times of the origami device are ~0.04 s and 0.1 s, respectively. The device height is ~120 mm. The recovery speed is estimated to be approximately 1.2 m/s. Basically, the overall energy generated in the pressing and releasing motions are comparatively the same. Therefore, the voltage amplitude generated in the pressing stage is obviously larger than that in the releasing stage. Figure 7c shows snapshots of a power generation demonstration in which light-emitting diodes (LEDs) are lit up by horizontal pressing and vertical tapping. Figure 7d, e show the output voltage of the W-TENG and an enlarged view for different operating frequencies in the horizontal direction, respectively. The flexible and deformable W-TENG can be easily integrated into shoes. Figure 7f shows an output performance demonstration in which the W-TENG is installed in shoes during walking and jogging motions.

### Water-proof capability characterization

**Fig. 8:**
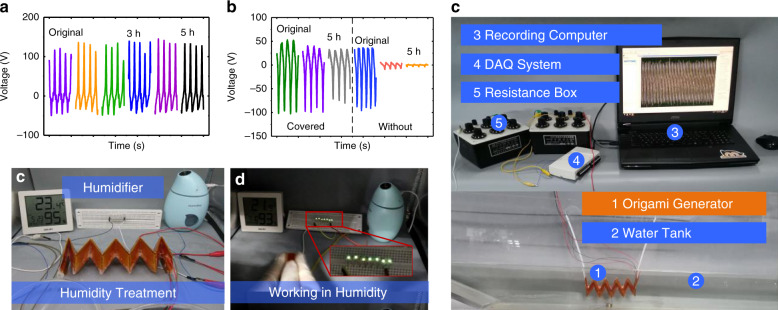
Water-proof characterization of the fabricated W-TENG device in moisture environments. **a** Power generation performance of the W-TENG after immersion treatment in water; **b** output performance comparison of two types of TENG devices: the proposed W-TENG with hermetically covered FEP electrets and the W-TENG with FEP electrets exposed to the outside; **c** experimental humid environment realized by a humidifier in closed chamber conditions; **d** snapshot demonstration of pressing the W-TENG to light up LEDs in the humid chamber; **e** experimental setup of immersing the fabricated W-TENG prototype in a water tank

Figure 8 shows the water-proof capability characterization of the proposed W-TENG for impulse excitation by hand tapping. Figure 8a shows the performance of the fabricated W-TENG in the room environment and after a soaking treatment in water for a period of time. The performance of the W-TENG device remains stable after long-term soaking treatment. Figure 8b shows a comparison of the output performances of the fabricated W-TENG device with hermetically covered FEP electrets and with FEP electrets exposed to a moisture environment. Supplementary material Video S2 shows a power demonstration of the fabricated W-TENG lighting up LEDs after immersion treatment in water. Figure 8c shows the experimental environment of a closed chamber with a humidity of up to 95%, which is created by an air humidifier. It can be clearly observed that the performance of the proposed W-TENG still remains stable even in the highly humid environment, while the performance of the TENG without protection abruptly drops. This further verifies the superiority of the water-proof capability of the newly proposed W-TENG device. Supplementary material Video S3 shows the operation of the fabricated W-TENG in the closed chamber with a highly humid environment. Figure 8d shows snapshots of lighting up LEDs by hand pressing the W-TENG device in the humid environment. Figure 8e shows the experimental setup of immersing the fabricated W-TENG prototype in a water tank.

## Conclusions

In summary, a Miura-origami-inspired electret/triboelectric power generator was conceptualized, fabricated and characterized for wearable energy harvesting with water-proof capability. Miura-origami folding, an ancient Japanese art of paper folding, has been successfully combined with the triboelectric energy conversion mechanism to create a new type of W-TENG device with excellent deformability, flexibility and stretchability. The thin-film FEP electrets have been hermetically sealed inside the Miura-origami-based “W-tube” structure, which is capable of separating them from the unfavorable environment outside. The corona discharge process has been employed to maximize the charge storage in the thin-film electrets and enhance the power generation. The performance of the proposed W-TENG device with different parameters has been intensively investigated. It has been found that with increasing number of energy generation units and careful control of the original height of the device, the performance can be largely enhanced. The power generation capabilities for different directions and forms of movement have been studied, including horizontal pressing, vertical tapping and lateral bending. The instantaneous open-circuit voltages for the horizontal and vertical directions can reach 791 V and 116 V with optimum powers of 691 μW at 50 MΩ and 220 μW at 35 MΩ, respectively. The charge stability has been further characterized in highly humid and closed environments, as well as after immersion treatment in a water tank. The experimental results demonstrate that good performance and stability have been obtained by the proposed W-TENG structure. The outcomes of this work represent the versatility and viability of the fusion of the art of origami and TENG techniques for broad application scenarios.

## Supplementary information

Video S1

Video S2

Video S3

Figure
