# Supplementary figures and images for: Miura-origami-inspired electret/triboelectric power generator for wearable energy harvesting with water-proof capability
Source: Microsyst Nanoeng. 2020 Aug 10;6:56. doi: 10.1038/s41378-020-0163-1 (PMC8433327; doi:10.1038/s41378-020-0163-1)

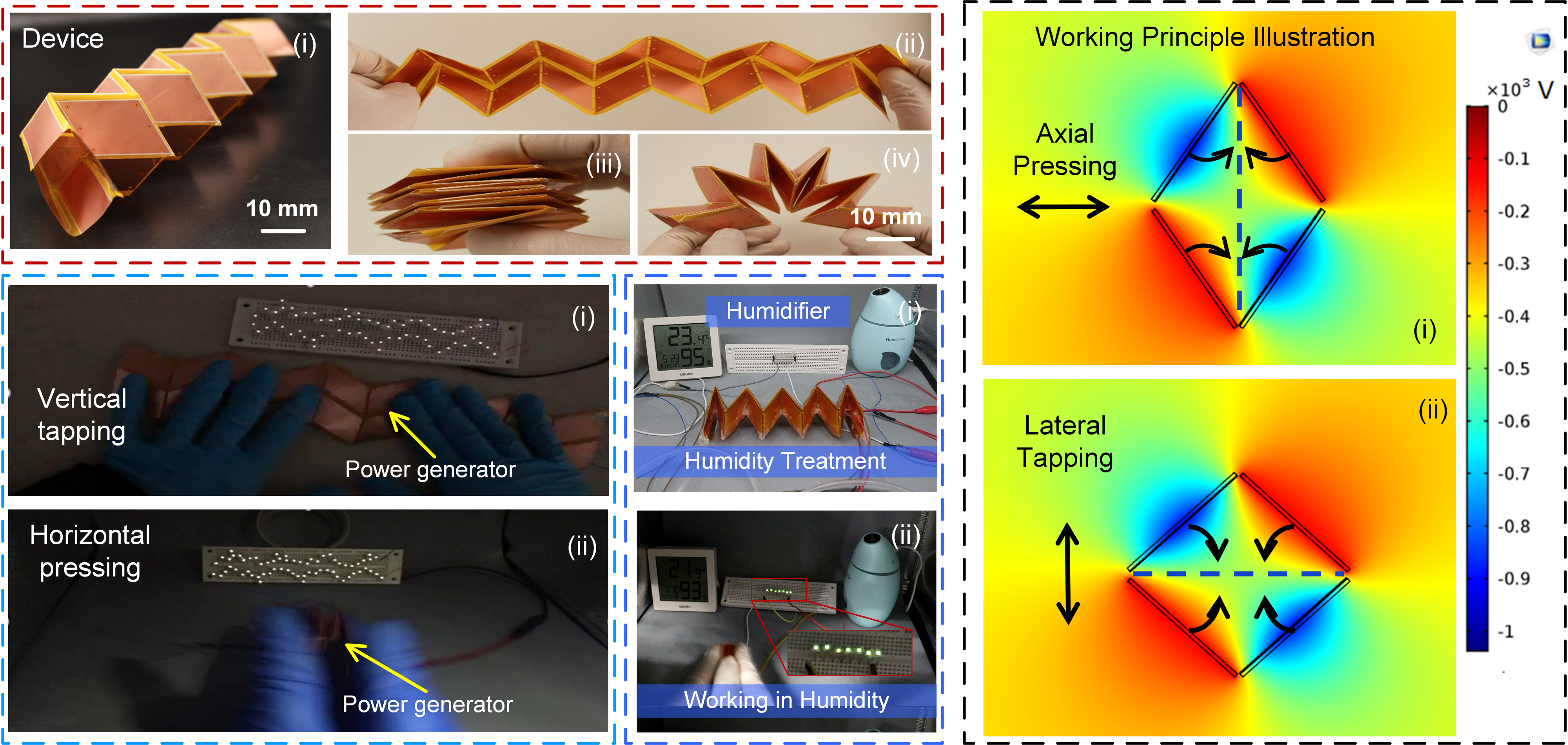

Supplement: Supplementary file 4 — Figure [file 41378_2020_163_MOESM4_ESM.tif]
